# Retraction Note: Long-term dynamics and distribution of large carnivores in Poland

**DOI:** 10.1038/s41598-026-52903-y

**Published:** 2026-05-12

**Authors:** Dorota Sienkiewicz-Paderewska, Jakub Paderewski

**Affiliations:** Department of Biometry, Warsaw University of Life Sciences, Nowoursynowska 159, 02-776 Warsaw, Poland

Retraction of: *Scientific Reports* 10.1038/s41598-025-30695-x, published online 29 November 2025

The Editors have retracted this Article.

After publication, concerns were raised regarding the large carnivore abundance data from the Polish Central Statistical Office (CSO) analysed in this study. According to the CSO [1], the abundance data is provided by organizational units of the State Forests or national parks that perform regional inventories not supported by a single, consistent, methodology. These inventories do not follow established standardised fieldwork methodologies for estimating large carnivore populations in Europe [2–4], which in turn affects the reliability and validity of the CSO data.

The Editors therefore no longer have confidence in the results and conclusions of this Article.

Both Authors disagree with the retraction.
